# Peripheral Macrophage-derived Exosomes promote repair after Spinal Cord Injury by inducing Local Anti-inflammatory type Microglial Polarization via Increasing Autophagy

**DOI:** 10.7150/ijbs.54302

**Published:** 2021-03-30

**Authors:** Baokun Zhang, Fangqi Lin, Jiqing Dong, Jingwen Liu, Zhenyu Ding, Jianguang Xu

**Affiliations:** 1 Department of Orthopedic Surgery, Shanghai Jiao Tong University Affiliated No. 6 People's Hospital, 600 Yishan Road, Shanghai 200233, China.; 2 Department of Orthopedic Surgery,Rizhao Hospital of Traditional Chinese Medicine.

**Keywords:** spinal cord injury, peripheral macrophage, exosomes, microglia polarization, autophagy

## Abstract

Treatment for spinal cord injury (SCI) remains a challenge worldwide, and inflammation is a major cause of secondary injury after SCI. Peripheral macrophages (PMs) have been verified as a key factor that exert anti-inflammatory effects after SCI, but the mechanism is unidentified. As local macrophages, microglia also exert significant effects after SCI, especially polarization. Exosomes show source cell-like biological functions to target cells and have been the subject of much research in recent years. Thus, we hypothesized the PM-derived exosomes (PM-Exos) play an important role in signal transmission with local microglia and can be used therapeutic agents for SCI in a series of *in vivo* and *in vitro* studies. For the *in vivo* experiment, three groups of Sprague-Dawley (SD) rats subjected to spinal cord contusion injury were injected with 200 µg/ml PM-Exos, 20 µg/ml PM-Exos or PBS via the tail vein. Recovery of the rats and of spinal cord function were observed. *In vitro*, we investigated the potential anti-inflammatory mechanism of PM-Exos and evaluated microglial autophagy, anti-inflammatory type microglia polarization and the upstream signaling pathway. The results showed that spinal cord function and recovery were better in the PM-Exo groups than the control group. In the *in vitro* study, microglial autophagy levels and the expression of anti-inflammatory type microglia were higher in the experimental groups than the control group. Moreover, the expression of proteins related to the PI3K/AKT/mTOR autophagic signaling pathway was suppressed in the PM-Exo groups. PM-Exos have a beneficial effect in SCI, and activation of microglial autophagy via inhibition of the PI3K/AKT/mTOR signaling pathway, enhancing the polarization of anti-inflammatory type microglia, that may play a major role in the anti-inflammatory process.

## Introduction

Treatment for spinal cord injury (SCI) and repair of the spinal cord are a challenge that clinicians face worldwide 1. SCI has a great impact on patients' quality of life and imposes a large burden on patients' families and society. Due to the insufficient regenerative and reparative abilities of the mature central nervous system (CNS), however, there are no satisfactory treatment strategies for SCI 2, 3. After SCI, the inflammatory environment around the lesion site is a key factor that promotes neuronal apoptosis, glial scar formation and degeneration of axons, thus inhibiting regeneration of the spinal cord 4, 5.

Recent research has shown that peripheral macrophages (PMs) effectively improve the anti-inflammatory microenvironment of the lesion site and are the key factors that promote repair after SCI 6. However, the mechanism of PMs at injured site after SCI is unknown, and the accumulation of PMs is complicated and controlled by a variety of factors, such as blockade of the blood-brain barrier. The function of microglia, s resident macrophages, is also important, and the polarization of microglia after SCI has been studied in recent years 7, especially the anti-inflammatory function of anti-inflammatory type microglia 8. Studies have revealed that neurons exposed to media conditioned by anti-inflammatory type microglia show improved survival 9. Do PMs have an effect on local microglial polarization at the injured site after SCI and what is the relationship between them?

**Figure 1:**
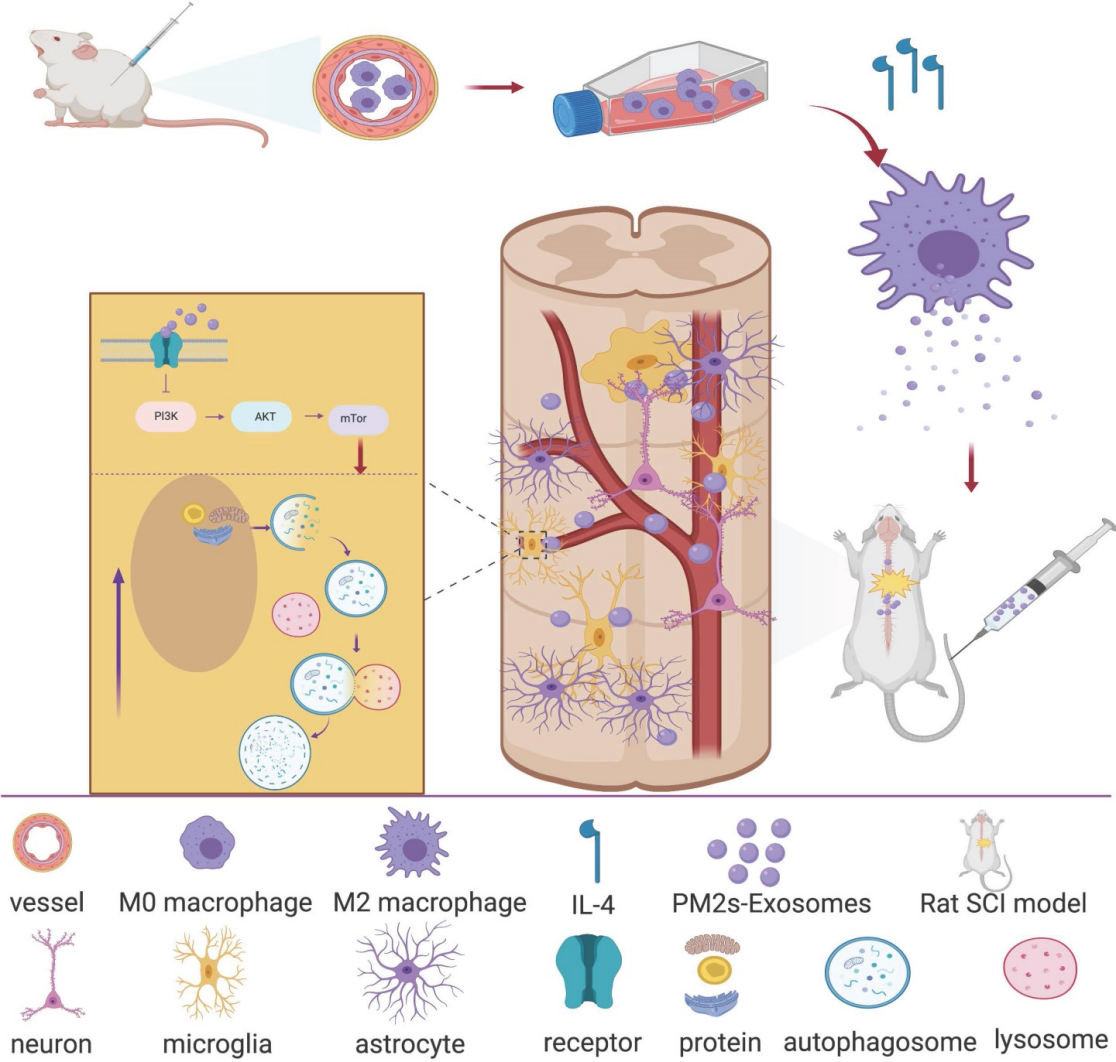
** Article scheme.** Peripheral macrophage-derived exosomes promote repair after spinal cord injury by increasing anti-inflammatory type microglial polarization via enhancing autophagy.

In recent years, research on the ability of exosomes to promote nerve repair has attracted much attention 10. Exosomes are vesicles that are secreted by cells and have a bilayer membrane and a diameter of 40-100 nm. Similar to the cells from which exosomes are derived, they mainly contain proteins, lipids, coding RNAs, noncoding RNAs, cytokines and growth factors. Exosomes can transmit biologically active substances and information about their source cells to target cells, thereby exerting cell-like biological functions, changing the microenvironment of effector cells and regulating the growth and production of target cells. This finding suggested that exosomes may exert important signal transmission effects after SCI, especially between PMs and microglia. Therefore, we hypothesize that PM-derived exosomes (PM-Exos) exert anti-inflammatory action at the lesion site by affecting the polarization of microglia, and we performed a series of *in vitro* and *in vivo* studies. The results were promising, and we conducted further studies to determine the underlying mechanism. We found that local microglial autophagy at the injured site plays a significant role in this process (Figure 1).

## Methods and Materials

### Study of exosomes extracted from PMs

#### Cell culture

Sprague-Dawley (SD) rats weighing 200-250 g were sacrificed. After being soaked in 75% alcohol, the rats were turned upside down, and 10 ml of serum-free DMEM culture medium was injected into the abdominal cavity. The abdominal cavity of each rat was opened after 10 min, and 10 ml of peritoneal fluid was extracted when the intestinal canal became pale yellow. The cells were then cultured with conventional DMEM, isolated and identified with CD11b.

#### Preparation of exosomes

Exosomes were extracted carefully from medium according to the method described by Thery et al. 11. Briefly, PMs were successively cultured with 10 μl of medium and serum-free medium at 37 °C for 24 h. After three washes with phosphate-buffered saline, the supernatant was collected and successively centrifuged at 300×g and 2000×g for 10 min to remove floating cells, dead cells and shedding vesicles. Next, the supernatant was collected and centrifuged at 10,000×g for 30 min to remove apoptotic bodies, exfoliated vesicles and cell debris. The supernatant was then collected, and the exosomes and contaminating proteins were precipitated by centrifugation at 140,000×g for 60 min in an ultra-high-speed centrifuge. Finally, the supernatant was discarded, and the pellet was resuspended in PBS and centrifuged at 140,000× g for 70 min to remove the contaminating proteins. The resulting precipitate was the PM-Exos.

### Identification of PM-Exos

#### Transmission electron microscopy (TEM)

The morphology of PM-Exos was examined using TEM. Briefly, 5 μl of isolated exosomes was placed onto carbon-Formvar-coated copper grids for 20 min. The grids were contrasted with 1% uranyl acetate for 20 s after being washed once with PBS and twice with distilled water. Finally, the grids were loaded and imaged with a transmission electron microscope (JEM-1400, JEOL, Japan) with an accelerating voltage set at 80 kV.

#### Dynamic light scattering (DLS) analysis

For determination of the size distribution of PMs-Exos, Nanosizer^TM^ technology (Malvern Instruments, Malvern, UK) was applied, and the particle size distribution was measured by DLS. Briefly, purified exosomes were resuspended in 1 ml of filtered PBS and slowly added to a Zetaview instrument (Particle Metrix, USA) for the detection, tracking and analysis of the Brownian motion of each particle. The particle diameter and concentration were analyzed.

### Western blotting

The PM-Exo-specific biomarkers CD9, CD63, CD81 and Tsg101 were analyzed by western blotting as previously reported 12. Briefly, a certain amount of isolated and purified exosomes was added to RIPA lysis buffer for extraction, and total protein was extracted from exosomes. The protein concentration was determined by the BCA method, and the protein was denatured by adding an appropriate ratio of loading buffer at 100 °C for 10 min. The PG110 and PG112 PAGE gel rapid preparation kits (Yazyme, China) were used to prepare 6% and 10% separation gels and concentrated gels for SDS-PAGE electrophoresis. Proteins were transferred to a PVDF membrane in transfer buffer, and the membrane was incubated with 5% skim milk powder, sealed and shaken for 1 h. A rabbit anti-mouse primary antibody was added, and the membrane was shaken at 4 °C overnight. After removal of the primary antibody and three washes with PBS for 10 min, the membrane was incubated with a horseradish peroxidase-labeled goat anti-mouse secondary antibody (1:5 000) for 1 h at room temperature. Finally, the secondary antibody was removed, and the membrane was washed three times with PBS (10 min each wash). ECL reagent was added for exposure and development with a chemiluminescence imaging system. The BCA protein data was normalized as per number of exosomes and an equal amount of protein was loaded.

### Cell uptake of PM-Exos

Exosomes were labeled with a PKH26 red fluorescent labeling kit according to the manufacturer's protocol, and they were then added to well-grown microglia at a concentration of 20 μg/ml and incubated at 37 °C for 8 h. Fixative was then added, and the exosomes were incubated in the dark for 10 min. DAPI was added, and the exosomes were incubated for an additional 10 min. Finally, PBS was added, and the uptake of exosomes by microglia was observed under a confocal microscope.

### Analysis of the effect of PM-Exos on polarization of microglia

BV2 cells (Chinese Academy of Sciences, Shanghai) were cultured in DMEM (Gibco, USA) and divided into the following three groups: control group, 20 μg/ml PM-Exo group and 200 μg/ml PM-Exo group. The 20 μg/ml PM-Exos group was treated with 20 μg/ml PM-Exos and medium, the 200 μg/ml PM-Exos group was treated with 200 μg/ml PM-Exos and medium, and the control group treated with PBS and medium only. The culture medium was changed once a day, and the control group did not receive any treatment.

### Identification of anti-inflammatory type microglia

#### Flow cytometry analysis

To determine microglia polarization status, according to the manufacturer's instructions, we labeled microglia with the LIVE/DEAD® Fixable Aqua Dead Cell Stain Kit. The cells were then washed with ice-cold FACS buffer (0.5% BSA/PBS) and centrifuged. The supernatant was removed, and the cells were incubated for 20 min on ice with an anti-mouse F4/80-eFluor 450 antibody (0.2 μg/100 μl; eBioscience), as a general marker of microglia, and a CD163 antibody (1 μg/100 μl; Sigma, USA), as a marker of anti-inflammatory type microglia.

### Quantitative polymerase chain reaction (qPCR)

Total RNA extraction was performed with TRIzol reagent (Invitrogen, USA) according the manufacturer's protocol, and SYBR Green reagent was used for qPCR to quantify the mRNA levels. The soluble mediators of anti-inflammatory type microglia (IL-10, CD206, Arg-1 and CD-163) were detected. GAPDH was used as a housekeeping gene, and the comparative ΔΔCT method was used to calculate the relative mRNA expression levels.

### Enzyme-linked immunosorbent assay (ELISA)

IL-10 levels in the cell supernatant were detected with an ELISA kit (Abcam, England) according to the manufacturer's instructions.

### *In vivo* study of a rat spinal cord contusion model

#### Surgical procedure, groups and treatment

All experimental rats were bred at the Laboratory Animal Center, and after being approved by the Animal Research Committee, the animal experiments were performed according to the regulations and guidelines of the Animal Ethics Committee. In total, twenty-four mature male SD rats (12 weeks old and mean body weight of 200-250 g) were used for this study. The surgical procedure was performed as described previously 13. Briefly, after the rats were anesthetized with 10% chloral hydrate, a laminectomy was performed at the 10^th^ thoracic vertebra (T10). A moderate contusion injury was induced by impacting the exposed dura of the spinal cord with a weight drop apparatus (10 g weight at a vertical height of 30 mm, 10 g × 30 mm). The animals with SCI were randomly divided into 3 groups of 6 rats each and treated with PBS, 20 μg/ml PM-Exos or 200 μg/ml PM-Exos via tail vein injection 30 min after SCI. Additionally, 6 rats were included as the sham group (laminectomy was performed but the spinal cord was not damaged). The wound of each rat was sutured. The rats were housed in individual cages, and the bladder of each rat was manually emptied twice a day until autonomic urination function was restored or the rats were sacrificed.

### Locomotion function assessment

On days 1, 3 and 7 after surgery, Basso, Beattie and Bresnahan (BBB) scores and the inclined plate test were used to evaluate the locomotor behavior of the rats. Two independent and well-trained investigators observed and assessed the locomotor function of rats as previously described. The final score of each rat was obtained by averaging the values from both investigators 14.

### Hematoxylin-eosin (H&E) staining and histological analysis

After tissue preparation, pathological sections were made. The sections were mounted on slides to dry. The paraffin was then removed, and the slides were washed with distilled water. The sections were stained with hematoxylin solution for several minutes. After dehydration in 70% and 90% alcohol for 10 min, the sections were stained with eosin for 2-3 min. The tissue was observed under an immunofluorescence microscope (Leica, Japan) and analyzed.

### Nissl staining and counting of motor neurons

Sections of the spinal cord were incubated in 0.1% Nissl staining solution (Abcam, England) for 3 min at 37 °C. After being rinsed with distilled water and dehydrated in 70, 80, 95% and 100% ethanol solutions, the sections were cleared in xylene for 5 min. The number of motoneurons in each section was observed under an immunofluorescence microscope (Leica, Japan). The average number of motoneurons per area was calculated.

### RNA extraction and quantitative real-time polymerase chain reaction (qRT-PCR)

A 1.5-cm piece of tissue was taken from the injured area for qRT-PCR. TRIzol reagent (Abcam, England) was used to extract total RNA, and reverse transcription of 1 mg of total RNA was performed with a reverse transcription mix (synthesized by Invitrogen). PCR results were analyzed using glyceraldehyde 3‐phosphate dehydrogenase (GAPDH) as an internal control. Quantification of gene expression for inflammatory cytokines was performed using the comparative cycle threshold.

### Enzyme‐linked immunosorbent assay (ELISA)

Rats were sacrificed 7 days after SCI, and tissue was collected from the lesion site to detect the levels of inflammation‐related cytokines. The levels of TNF-α, IL‐10, IL‐6 and IL‐1β were detected using ELISA kits (Abcam, England).

### Proteome profiler

The expression of cytokines in the rat spinal cord was measured in 100 μg protein per sample with a Rat Cytokine Array Profiler (R&D System, USA) according to the manufacturer's instructions. Spot size was analyzed, and the results are expressed as the fold change in the exosome groups compared to the PBS and sham groups.

### Anterograde tracing of axons

Neurobiotin 350 (10%, 10,000 MW; Vector, USA) was used for axon tracing. Briefly, 2 weeks after SCI, Neurobiotin 350 was injected at 4 sites around T2 of the rats after anesthesia and fixation. Two weeks later, the rats were sacrificed, and a 1.5-cm piece of tissue was taken from the damaged area after formaldehyde perfusion. Finally, the pathological sections were generated, and the axons were observed under an immunofluorescence microscope.

### Detection of autophagy

#### TEM

Cells were pelleted by centrifugation and resuspended three times in PBS. A 2.5% glutaraldehyde fixative solution was added, and the cells were fixed for 3 h after the supernatant was removed. After three washes with PBS, the cells were shaken and fixed with 0.5% osmic acid for an additional 3 h. After embedding and double staining with uranyl acetate/lead citrate, the sections were visualized at 60 kV under H-7650 TEM (JEOL1400).

### Tandem stubRFP-sensGFP-LC3 confocal microscopy

StubRFP-sensGFP-LC3 transfected BV2 cells were incubated into cell culture dishes at a density of 1×10^5^ cells per culture dish. After being washed three times with PBS, the cells were placed on a laser-scanning confocal microscope (Nikon, Japan) for observation. The number of autolysosomes and autophagosome per field of view was observed in 20 randomly selected fields of view.

### Western blot

After total protein lysates were obtained with RIPA buffer, the BCA method was applied to measure the protein concentration. After the membranes were incubated with the corresponding primary and secondary antibodies, the signals were detected with an ECL assay kit (Amersham, Sweden).

### Statistical analysis

All values in the figures and text were from three independent experiments. All results are shown as the mean ± SEM. Statistical analysis were performed with GraphPad Prism V8.0 (San Diego, CA, USA). Student's t test was used to analyze the difference between the two groups, and the one-way ANOVA was used to analyze the difference among two or more groups. The results of the cued conditional response were analyzed using two-way ANOVA. Post-hoc correction was also performed for multiple comparisons between groups over time. Differences were considered significant at P < 0.05.

## Results

### Characterization of exosomes

**Figure 2:**
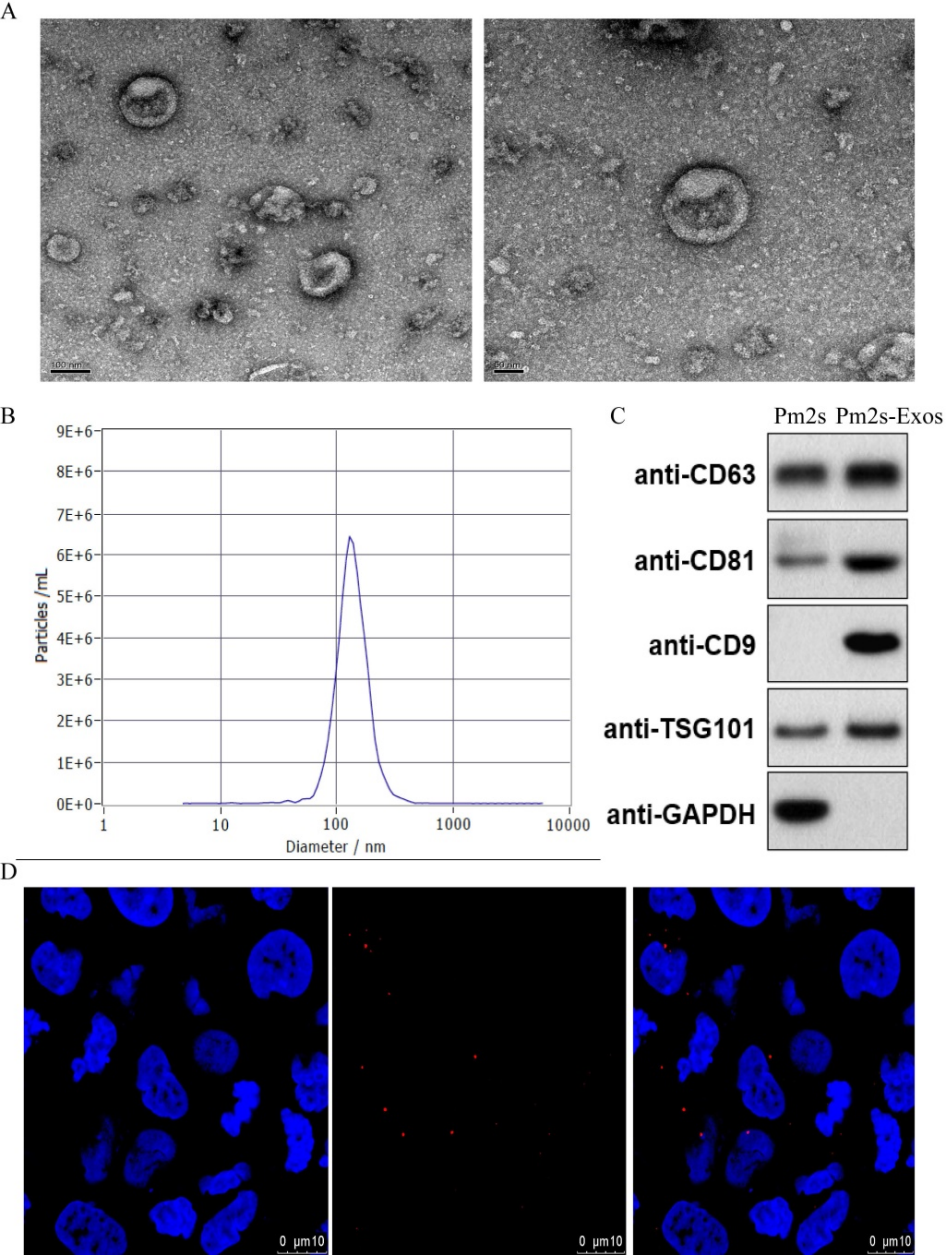
** Identification of Exosomes. (A)** Morphology of exosomes observed by TEM. Scale bar: 100 nm/50 nm **(B)** Particle size distribution of exosomes measured by DLS. **(C)** Exosome surface markers (CD81, CD9, CD63 and TSG-101) were measured using western blotting.** (D)** Representative immunofluorescence photomicrograph of PKH26 (red)-labeled exosomes absorbed by cells. Nuclei were stained by DAPI (blue).

TEM images were used to observe the general morphology of the exosomes isolated from PMs, and the results showed that the majority of these particles exhibited a round or cup-shaped morphology. According to DLS analysis, the size of most particles ranged from 50 to 150 nm, indicating that they were exosomes. Moreover, western blotting revealed that the cells expressed representative exosome surface markers. Exosomes were labeled with the exosome marker, PKH26, and incubated with microglia to detect the phagocytosis of exosomes by microglia. After observation under a confocal microscope, the cells were further confirmed to be exosomes (Figure 2).

### PM-Exos promote anti-inflammatory type microglial polarization

**Figure 3:**
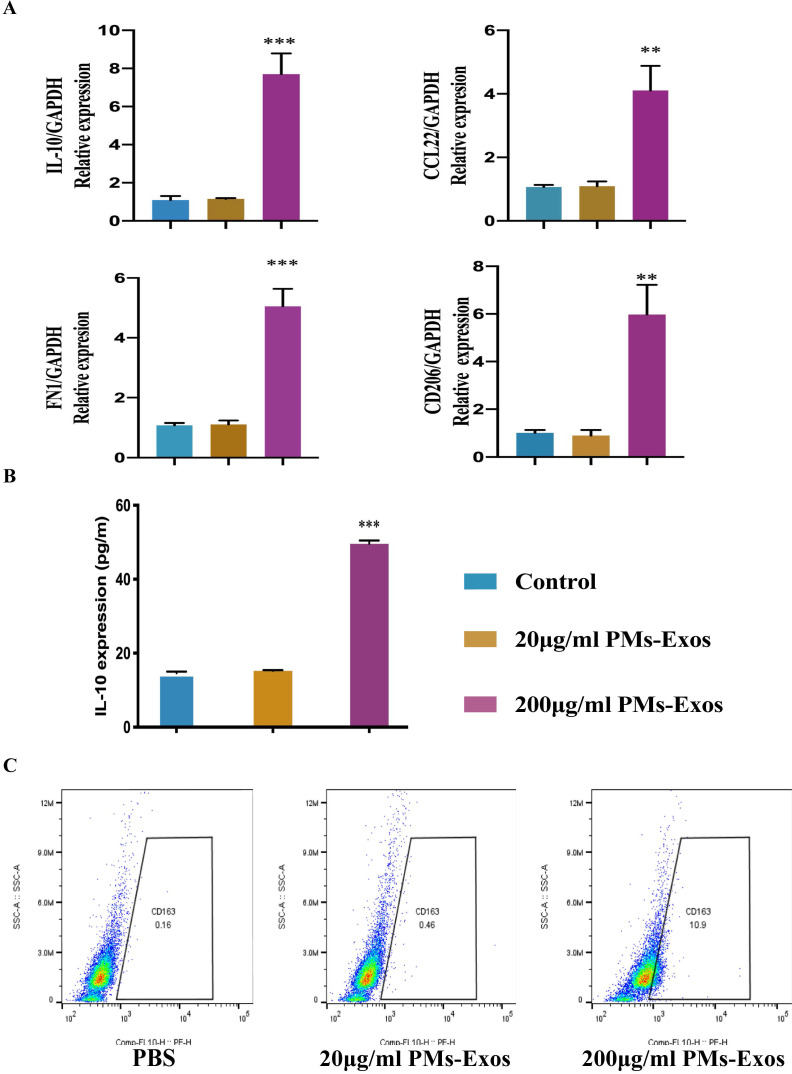
** Identification of anti-inflammatory type microglia. (A)** Representative relative mRNA expression levels of M2 macrophages. This experiment was repeated independently three times. **P<0.01 and ***P<0.001 compared to the control groups.** (B)** Enzyme‐linked immunosorbent assay (ELISA) to measure IL-10. ***P<0.001 compared to the control groups.** (C)** Flow cytometry analysis of anti-inflammatory type microglia.

The ELISA results showed that the expression of IL-10 was higher in the PM-Exo-treated cells than in the PBS-treated cells. PCR revealed that the RNA expression of CD206, CD163, Arg-1 and IL-10 in the PM-Exo-treated group was also higher than that in the control groups, especially in the 200 μg/ml PM-Exo-treated group, indicating that the cells were anti-inflammatory type microglia and there was a positive effect of the PM-Exos on M2 polarization. Moreover, flow cytometry showed that there were more anti-inflammatory type microglia in the 200 μg/ml PM-Exo-treated group than in the other groups, confirming that the cells were anti-inflammatory type microglia (Figure 3).

### PM-Exos improve functional recovery after SCI

**Figure 4:**
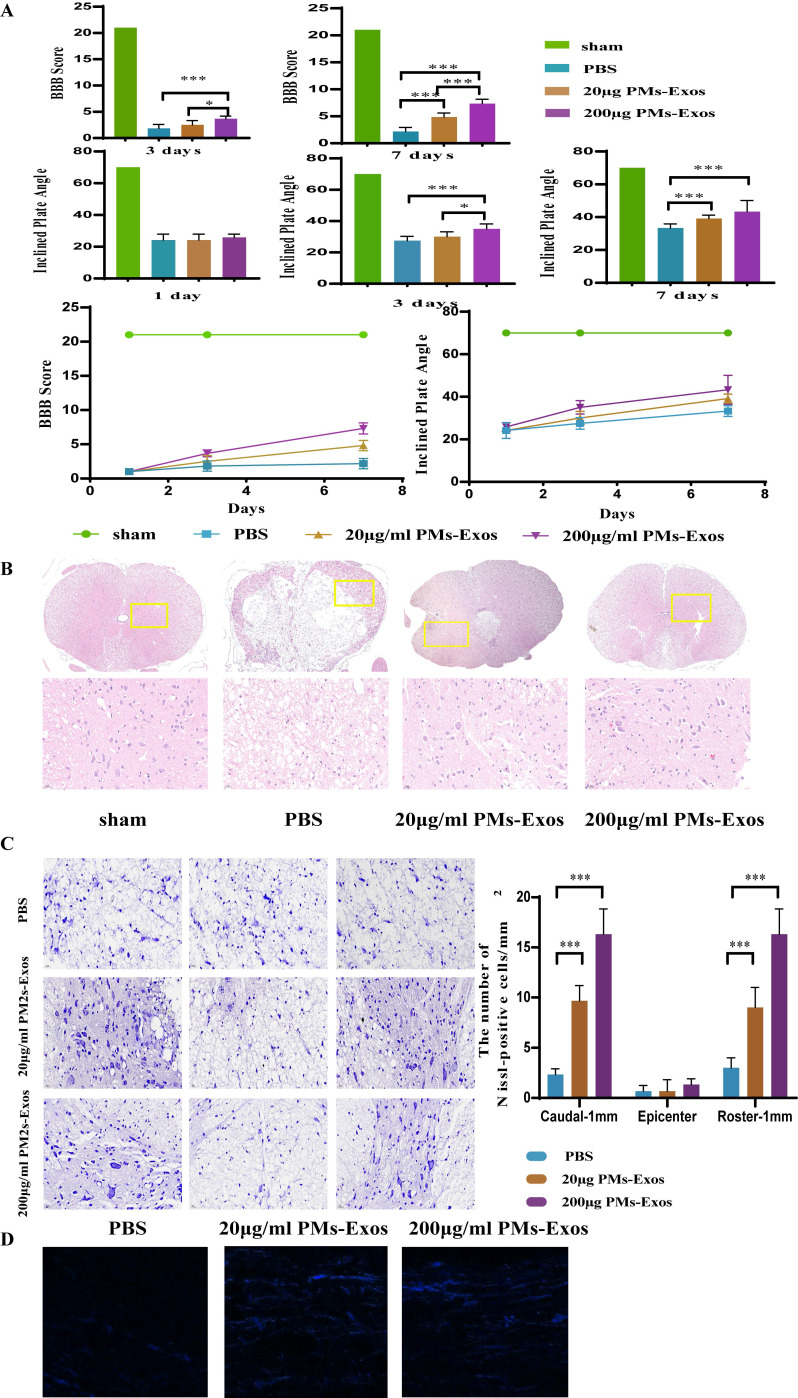
**The positive effect of PM-Exos on spinal cord injured rats. (A)** BBB score and inclined plate test were performed, and the results are shown. *P <0.05, **P<0.01 and ***P<0.001** (B)** Hematoxylin-Eosin (HE) staining and histological analysis. **(C)** Nissl staining and cell counting of motor neurons. **(D)** Anterograde tracing of axons. ***P<0.001.

On days 1, 3 and 7 after SCI, the locomotor function of the animals was observed by two well-trained observers. As shown in Figure 4, the differences in BBB scores and inclined plate test performance among the three groups were not significant on day 1. On day 3, however, the rats in the 200 μg/ml PM-Exo group performed better than those in the other two groups. On day 7 after SCI, the average BBB score and performance in the inclined plate test of both the 20 μg/ml and 200 μg/ml PM-Exo groups were better than those of the PBS group (Figure 4).

### PM-Exos promote tissue repair after SCI

H&E staining and Nissl staining were used to observe the morphology of the spinal cord. As shown in Figure 4, damage to the central gray matter and dorsal white matter was visualized by H&E staining, and this damage was more pronounced in the PBS group than in the PM-Exo groups. Moreover, H&E staining showed less tissue damage in the 200 μg/ml PM-Exo group than in the 20 μg/ml PM-Exo group. Furthermore, Nissl staining was used to determine the number of spinal neurons. There were more Nissl-positive cells in the anterior horn of the spinal cord in the 200 μg/ml PM-Exo group than in the 20 μg/ml PM-Exo group and the PBS group. For axon tracing, quantitative analysis showed that the number of Neurobiotin 350-positive fibers was significantly increased in the 200 μg/ml PM-Exo group and the 20 μg/ml PM-Exo group. These findings suggested that a high concentration of PM-Exos protects damaged neurons and reduces the effects of SCI.

### PM-Exos attenuate the expression of inflammatory cytokines after SCI

**Figure 5:**
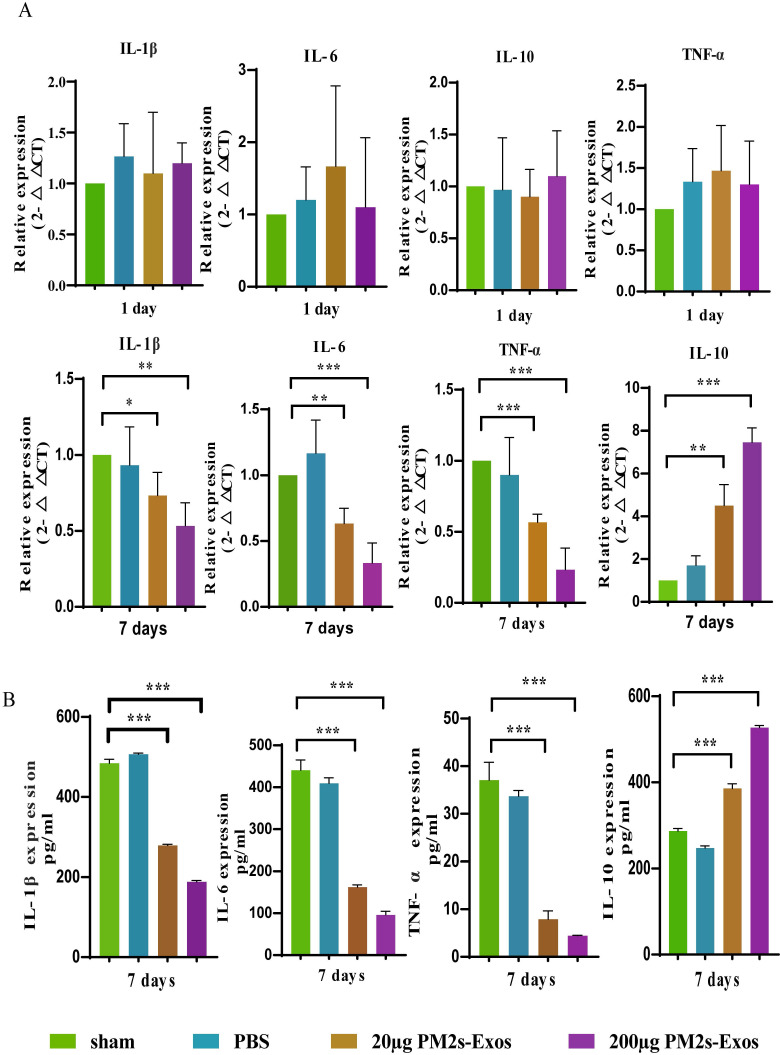
** PM-Exos attenuate the expression of inflammatory cytokines after SCI. (A)** The representative qPCR expression of inflammatory cytokines. *P <0.05, **P<0.01 and ***P<0.001** (B)** Enzyme‐linked immunosorbent assay (ELISA) of inflammatory protein. *P <0.05, **P<0.01 and ***P<0.001.

**Figure 6:**
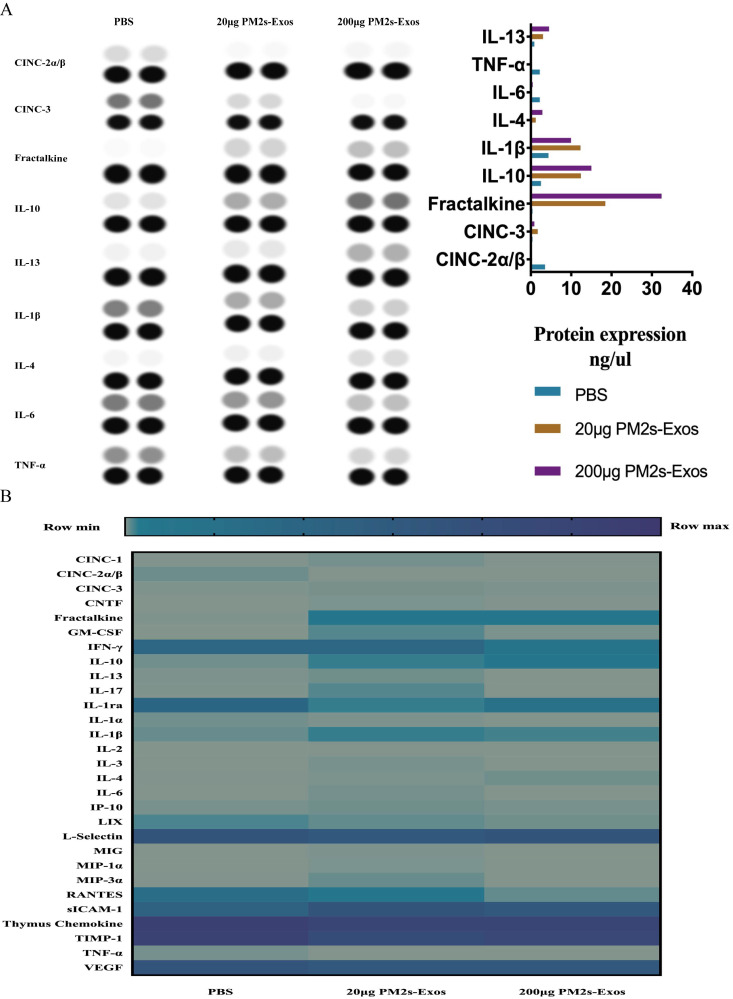
** Proteome profiler of inflammatory cytokines after SCI. (A)** The proteome profiler of inflammatory cytokines was performed, and the analyzed results are shown.** (B)** Thermography of inflammatory cytokines comparison of the three groups.

As shown in Figure 5, the qPCR results showed no significant differences in inflammatory cytokine expression among the three groups on day 1 after SCI, and the same results were obtained with ELISA. On day 7, however, the expression of proinflammatory cytokines was significantly decreased in the PM-Exo groups, especially in the 200 μg/ml PM-Exo group, compared to the PBS group. There were also differences in the anti-inflammatory activity in each group with the expression of IL-6 being much higher in the PM-Exo group than in the PBS group. The proteome profiler array also revealed differences in the expression of proinflammatory cytokines and anti-inflammatory cytokines as shown in Figure 6.

### Stimulation of microglia with PM-Exos promotes and induces autophagy

**Figure 7:**
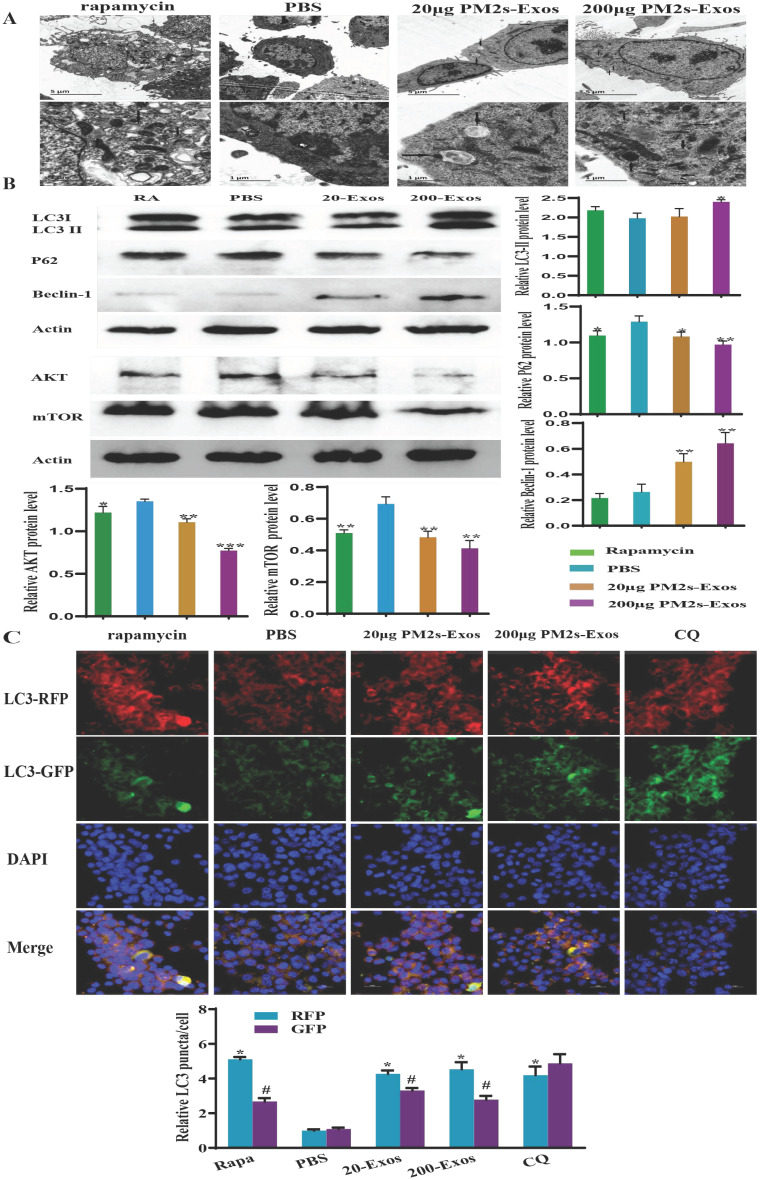
** PM-Exos induce microglial autophagy via downregulation of the AKT/mTOR signaling pathway. (A)** Transmission electron microscopy analysis of microglia. The arrows indicate autophagosomes. Scale bars: 5 µm (upper) and 2 µm (lower). **(B)** Western blot analysis results of autophagy- and AKT/mTOR signaling pathway-related proteins.** (C) stubRFP-sensGFP-LC3-infected rat spinal cord microglia for the autophagy study.** In BV2 cells, rapamycin and PM-Exos promoted the occurrence of autophagy flux (red fluorescence was enhanced and green fluorescence was weakened due to the formation of autolysosomes). Chloroquine (CQ) inhibited the formation of autolysosomes. Therefore, when the red fluorescence was enhanced, the green fluorescence was enhanced at the same time. The difference was statistically significant. *vs. PBS group, P<0.05; #vs. RFP, P<0.05.

To detect the effect of PM-Exos on microglial autophagy, we examined the protein levels of autophagy-related proteins, including LC3-II/Ⅰ, Beclin-1 and p62, by western blotting. As shown in Figure 7, the LC3-II/Ⅰ level was increased in the 200 μg/ml PM-Exo group. The same trend was also found for Beclin-1, but the variation in expression of p62 was in the opposite direction. These results indicated that microglial autophagy was induced in the PM-Exo groups. The tandem stubRFP-sensGFP-LC3 method was then used to observe the occurrence of autophagic flux. The number of autolysosomes and early stage autophagosomes in the PM-Exo groups were notably higher than those in the PBS group. Moreover, TEM also indicated that there were more autophagosomes in the PM-Exo groups than in the PBS group.

### The PI3K/AKT/mTOR autophagic signaling pathway is downregulated

To investigate the potential mechanism by which microglial autophagy is activated, western blotting was performed to detect the expression of AKT and mTOR, which are involved in the classical autophagy pathway. Compared to the PBS group, the expression of these proteins in the 200 μg/ml PM-Exo group was significantly decreased (Figure 7).

## Discussion

In the present study, we investigated one way that PMs affect repair after SCI, and we used PM-Exos to intervene in the early stage of SCI for the first time. We verified the relationship between PMs and local microglia after SCI, and to explore the potential mechanism of this beneficial effect, we made reasonable inferences from recent studies showing that autophagy may play a significant role in the process. We performed a series of *in vitro* and *in vivo* studies and proved that the protective effect of PM-Exos, which involves increasing anti-inflammatory type microglial polarization by enhancing autophagy via activation the PI3K/AKT/mTOR pathway, after SCI injury is an important factor for neuronal protection.

SCI is a worldwide problem, and the burden that SCI imposes on society and families is devastating. Additionally, treating SCI has long been a challenge for spinal surgeons. A series of biochemical events occur after SCI-mediated secondary injury 15, and infiltration of resident macrophages and microglia plays a significant role in this process. The proinflammatory and anti-inflammatory effects of macrophages and microglia play critical roles throughout the secondary injury processes. However, a recent study revealed that the recruitment of PMs is essential for successful spinal cord regeneration 6. In our study, to explore the effect that PMs have on local microglia polarization, we extracted monocytes from the rat abdominal cavity and differentiated them into macrophages. We focused on exerting anti-inflammatory effects at the SCI site.

Exosomes, which are small (40-100 nm) membranous vesicles, carry various biologically active molecules, including coding RNAs, noncoding RNAs, DNA, antigen presentation molecules and proteins 16-18. Thus, exosomes regulate communication between cells and the transmission of pathogens 1, 19. More importantly, exosomes derived from different cell types have different characteristics that reflect the type and activation state of their mother cells 20. The results of our study confirmed the above findings. The *in vivo* study clearly indicated that PMs had anti-inflammatory effects, and the PCR, ELISA and proteome profiler results showed that the PM-Exos, especially at a higher concentration, had a significantly greater effect than the control. As expected, exosomes derived from PMs exerted anti-inflammatory effects similar to those of their mother cells. Observation of the experimental rats as well as Nissl staining and H&E staining of the spinal cord also showed that the PM-derived exosomes improved functional recovery after SCI. Further, attenuation of inflammation may play a significant role in this process. However, it remained unknown what PMs stimulate or activate.

Microglia are glial cells that are located throughout the spinal cord, and they compromise approximately 10%-15% of all CNS cells 21. As resident macrophage cells, microglia play a significant role in many nervous system diseases, and they are activated and dominating drivers of the response to SCI 22. The study of polarization of microglia has always been a hotspot 23, 24. Studies have shown that the M2 polarization of microglia is beneficial for local anti-inflammatory effects after SCI 25. In the present research, we found that with the increasing number of anti-inflammatory type microglia, the inflammatory indicators decreased sharply, indicating the anti-inflammatory effect of anti-inflammatory type microglia. The *in vivo* study also indicated that in the PM group, with more polarization of anti-inflammatory type microglia, the recovery of SCI rats was better than in other groups.

Recent research has shown that autophagy, as a vital intracellular degradation process, plays a critical role after neurological diseases by regulating microglia polarization 26. It has been observed that markers of autophagy are increased in microglia after SCI 22. Although the mechanism by which autophagy exerts an effect on microglia is currently unclear, the activation of microglia by autophagy after SCI is dynamic, changing with time and the number microglia 27-29. In general, anti-inflammatory properties are associated with high levels of autophagic flux, whereas proinflammatory properties are associated with inhibition of autophagic flux 29. In the present study, to investigate whether the anti-inflammatory type microglia polarization and anti-inflammatory environment was related to microglial autophagy, we performed a series of *in vitro* studies. The results showed that in the PM groups, the number of autophagosomes and autophagic flux were significantly increased compared to those in the control group, which is consistent with previous studies.

The signaling pathway that controls autophagy is complex, and the PI3K/AKT/mTOR signaling pathway is one of the pathways that is most closely related to autophagy 30. There are two mTOR complexes, and mTORC1 is a critical negative regulator of autophagy 31, 32. The PI3K/AKT/mTOR pathway is a major upstream modulator of autophagy. In the present study, we found that AKT and mTOR were decreased significantly in the PM-Exo groups compared to the control group as determined by western blotting. Therefore, we concluded that suppression of the PI3K/AKT/mTOR pathway enhances autophagy in microglia.

## Conclusion

In summary, we aimed to treat SCI, one of the most challenging conditions for clinicians and society, by combining anti-inflammatory PMs and exosomes. By performing an *in vivo* study in a rat model of spinal cord contusion injury and a series of *in vitro* studies, we found that treating SCI with PM-Exos is promising and that the anti-inflammatory effect of PM-Exos is clearly beneficial for recovery after SCI. We investigated the potential mechanism of this therapeutic effect, and we found that downregulation of the PI3K/AKT/mTOR signaling pathway is a key factor that activates and promotes autophagy in microglia, thus increasing anti-inflammatory type microglial polarization and stimulating the anti-inflammatory properties of local microglia. Our study demonstrated that therapeutic strategies involving PM-Exos have great potential for recovery from SCI.

## Abbreviations

SCI: spinal cord injury
PMs: peripheral macrophages
PM-Exos: peripheral macrophages-derived exosomes
DLS: dynamic light scattering
TEM: transmission electron microscopy
BBB score: Basso, Beattie and Bresnahan score
ELISA: Enzyme‐linked immunosorbent assay
GAPDH: glyceraldehyde 3‐phosphate dehydrogenase

## References

[1] Tsai MJ, Liou DY, Lin YR, Weng CF, Huang MC, Huang WC (2018). Attenuating Spinal Cord Injury by Conditioned Medium from Bone Marrow Mesenchymal Stem Cells. J Clin Med.

[2] Seo DK, Kim JH, Min J, Yoon HH, Shin ES, Kim SW (2017). Enhanced axonal regeneration by transplanted Wnt3a-secreting human mesenchymal stem cells in a rat model of spinal cord injury. Acta Neurochir (Wien).

[3] Yin H, Shen L, Xu C, Liu J (2018). Lentivirus-Mediated Overexpression of miR-29a Promotes Axonal Regeneration and Functional Recovery in Experimental Spinal Cord Injury via PI3K/Akt/mTOR Pathway. Neurochem Res.

[4] Kjell J, Olson L (2016). Rat models of spinal cord injury: from pathology to potential therapies. Dis Model Mech.

[5] Liu S, Sandner B, Schackel T, Nicholson L, Chtarto A, Tenenbaum L (2017). Regulated viral BDNF delivery in combination with Schwann cells promotes axonal regeneration through capillary alginate hydrogels after spinal cord injury. Acta Biomater.

[6] Tsarouchas TM, Wehner D, Cavone L, Munir T, Keatinge M, Lambertus M (2018). Dynamic control of proinflammatory cytokines Il-1beta and Tnf-alpha by macrophages in zebrafish spinal cord regeneration. Nat Commun.

[7] Xu S, Zhu W, Shao M, Zhang F, Guo J, Xu H (2018). Ecto-5'-nucleotidase (CD73) attenuates inflammation after spinal cord injury by promoting macrophages/microglia M2 polarization in mice. J Neuroinflammation.

[8] Han D, Yu Z, Liu W, Yin D, Pu Y, Feng J (2018). Plasma Hemopexin ameliorates murine spinal cord injury by switching microglia from the M1 state to the M2 state. Cell Death Dis.

[9] Kigerl KA, Gensel JC, Ankeny DP, Alexander JK, Donnelly DJ, Popovich PG (2009). Identification of two distinct macrophage subsets with divergent effects causing either neurotoxicity or regeneration in the injured mouse spinal cord. J Neurosci.

[10] Guo S, Perets N, Betzer O, Ben-Shaul S, Sheinin A, Michaelevski I (2019). Intranasal Delivery of Mesenchymal Stem Cell Derived Exosomes Loaded with Phosphatase and Tensin Homolog siRNA Repairs Complete Spinal Cord Injury. ACS Nano.

[11] Thery C, Amigorena S, Raposo G, Clayton A (2006). Isolation and characterization of exosomes from cell culture supernatants and biological fluids. Curr Protoc Cell Biol.

[12] Singhto N, Kanlaya R, Nilnumkhum A, Thongboonkerd V (2018). Roles of Macrophage Exosomes in Immune Response to Calcium Oxalate Monohydrate Crystals. Front Immunol.

[13] Hu JZ, Huang JH, Zeng L, Wang G, Cao M, Lu HB (2013). Anti-apoptotic effect of microRNA-21 after contusion spinal cord injury in rats. J Neurotrauma.

[14] Wyatt LA, Keirstead HS (2012). Stem cell-based treatments for spinal cord injury. Prog Brain Res.

[15] Zhang B, Gensel JC (2014). Is neuroinflammation in the injured spinal cord different than in the brain? Examining intrinsic differences between the brain and spinal cord. Exp Neurol.

[16] Balaj L, Lessard R, Dai L, Cho YJ, Pomeroy SL, Breakefield XO (2011). Tumour microvesicles contain retrotransposon elements and amplified oncogene sequences. Nat Commun.

[17] Luketic L, Delanghe J, Sobol PT, Yang P, Frotten E, Mossman KL (2007). Antigen presentation by exosomes released from peptide-pulsed dendritic cells is not suppressed by the presence of active CTL. J Immunol.

[18] Taylor DD, Gercel-Taylor C (2008). MicroRNA signatures of tumor-derived exosomes as diagnostic biomarkers of ovarian cancer. Gynecol Oncol.

[19] Zhang ZG, Buller B, Chopp M (2019). Exosomes - beyond stem cells for restorative therapy in stroke and neurological injury. Nat Rev Neurol.

[20] Xu R, Rai A, Chen M, Suwakulsiri W, Greening DW, Simpson RJ (2018). Extracellular vesicles in cancer - implications for future improvements in cancer care. Nat Rev Clin Oncol.

[21] Su P, Zhang J, Wang D, Zhao F, Cao Z, Aschner M (2016). The role of autophagy in modulation of neuroinflammation in microglia. Neuroscience.

[22] Loane DJ, Byrnes KR (2010). Role of microglia in neurotrauma. Neurotherapeutics.

[23] Milich LM, Ryan CB, Lee JK (2019). The origin, fate, and contribution of macrophages to spinal cord injury pathology. Acta Neuropathol.

[24] Zhang Y, Liu Z, Zhang W, Wu Q, Zhang Y, Liu Y (2019). Melatonin improves functional recovery in female rats after acute spinal cord injury by modulating polarization of spinal microglial/macrophages. J Neurosci Res.

[25] Fan H, Tang HB, Shan LQ, Liu SC, Huang DG, Chen X (2019). Quercetin prevents necroptosis of oligodendrocytes by inhibiting macrophages/microglia polarization to M1 phenotype after spinal cord injury in rats. J Neuroinflammation.

[26] Jin MM, Wang F, Qi D, Liu WW, Gu C, Mao CJ (2018). A Critical Role of Autophagy in Regulating Microglia Polarization in Neurodegeneration. Front Aging Neurosci.

[27] Cadwell K, Liu JY, Brown SL, Miyoshi H, Loh J, Lennerz JK (2008). A key role for autophagy and the autophagy gene Atg16l1 in mouse and human intestinal Paneth cells. Nature.

[28] Francois A, Terro F, Janet T, Rioux Bilan A, Paccalin M, Page G (2013). Involvement of interleukin-1beta in the autophagic process of microglia: relevance to Alzheimer's disease. J Neuroinflammation.

[29] Sil P, Muse G, Martinez J (2018). A ravenous defense: canonical and non-canonical autophagy in immunity. Curr Opin Immunol.

[30] Wang Z, Zhou L, Zheng X, Chen G, Pan R, Li J (2017). Autophagy protects against PI3K/Akt/mTOR-mediated apoptosis of spinal cord neurons after mechanical injury. Neurosci Lett.

[31] Cuyas E, Corominas-Faja B, Joven J, Menendez JA (2014). Cell cycle regulation by the nutrient-sensing mammalian target of rapamycin (mTOR) pathway. Methods Mol Biol.

[32] Schmelzle T, Hall MN (2000). TOR, a central controller of cell growth. Cell.

